# Identifying Preferred Features of Influenza Vaccination Programs Among Chinese Clinicians Practicing Traditional Chinese Medicine and Western Medicine: Discrete Choice Experiment

**DOI:** 10.2196/63314

**Published:** 2025-01-20

**Authors:** Liuren Zhang, Linchen Chu, Maria E Sundaram, Yi Zhou, Xiu Sun, Zheng Wei, Chuanxi Fu

**Affiliations:** 1 Institute of Infectious Disease and Vaccine, School of Public Health, Zhejiang Chinese Medical University, Hangzhou, China; 2 The Third People's Hospital of Hubei Province, Wuhan, China; 3 Center for Clinical Epidemiology and Population Health, Marshfield Clinic Research Institute, Marshfield, WI, United States; 4 The Third Affiliated Hospital of Zhejiang Chinese Medical University, Hangzhou, China; 5 The Second Affiliated Hospital of Zhejiang University School of Medicine, Hangzhou, China

**Keywords:** influenza vaccination program, traditional Chinese medicine, clinicians, vaccine, health care worker, hospital-acquired, effectiveness, antiviral, cross-sectional study

## Abstract

**Background:**

Achieving high vaccine coverage among clinicians is crucial to curb the spread of influenza. Traditional Chinese medicine (TCM), rooted in cultural symbols and concepts without direct parallels in modern Western medicine, may influence perspectives on vaccination. Therefore, understanding the preferences of TCM clinicians towards influenza vaccines is of great importance.

**Objective:**

To understand preferences for features of influenza vaccination programs and identify the optimal influenza vaccination program among clinicians practicing TCM and Western medicine.

**Methods:**

We conducted a discrete choice experiment with a national sample of 3085 Chinese clinicians from various hospital levels (n=1013 practicing TCM) from January to May 2022. Simulations from choice models using the experimental data generated the coefficients of preference and predicted the uptake rate of different influenza vaccination programs. Clinicians were grouped by vaccine preference classification through a latent class analysis.

**Results:**

All included attributes significantly influenced clinicians’ preferences for choosing an influenza vaccination program. An approximate hypothetical 60% increase of vaccine uptake could be obtained when the attitude of the workplace changed from “no notice” to “encouraging of vaccination”; there was an approximate hypothetical 35% increase of vaccine uptake when vaccination campaign strategies changed from “individual appointment” to “vaccination in a workplace setting.” In the entire sample, about 30% (946/3085) of clinicians preferred free vaccinations, while 26.5% (819/3085) comprehensively considered all attributes, except vaccination campaign strategies, when making a decision about choosing an influenza vaccination program. Clinicians who practiced TCM, worked in tertiary hospital, or had at least a postgraduate degree exhibited a lower preference for free vaccinations. Clinicians who practiced Western medicine, worked in primary hospital, or had at most a bachelor’s degree had a higher preference for vaccinations in workplace settings.

**Conclusions:**

Offering a range of influenza vaccination programs targeting the preferred attributes of different clinician groups could potentially encourage more clinicians, including those practicing TCM, to participate in influenza vaccination programs.

## Introduction

Annual seasonal influenza epidemics result in 290,000-650,000 deaths worldwide and over 88,000 preventable deaths in China annually [1,2]. During influenza seasons, health care workers (HCWs) are at a high risk of infection, leading to economic loss for the institutions they work for and potentially causing staff shortages [3]. HCWs may be asked or pressured to work despite having an influenza infection, contributing to clinic- or hospital-acquired influenza transmission [4]. Previous studies have indicated that clinicians play a crucial role in influencing vulnerable groups. Clinicians who were vaccinated were more likely to recommend vaccinations to their patients, highlighting the critical role of this group in improving vaccine uptake [5]. Influenza vaccination is the most cost-effective measure to protect high-risk groups against severe influenza-associated diseases and deaths, and the World Health Organization recommends the influenza vaccination in particular for specific target groups, including HCWs [6].

However, despite efforts to promote influenza vaccination among HCWs, uptake rates remain unsatisfactory in China. In the 2019-2020 season, only 61% of Chinese clinicians, who were recruited at a platform for respiratory medical professionals, received vaccines, which was lower than the 87.9% in Finland [7,8]. In the 2021-2022 season, the coverage was even higher in the United States, which had 91.3% clinicians who received vaccines [9]. Barriers to influenza vaccine uptake among HCWs in China include out-of-pocket costs and insufficient workplace regulations [7]. For example, workplaces did not require or encourage clinicians to get vaccinated before the flu season. Most importantly, previous studies have primarily treated Chinese clinicians as practitioners of modern medicine, overlooking the significant presence of clinicians practicing traditional Chinese medicine (TCM) in China [7,10].

Modern Western medicine, originating in Europe, dominates medical practices globally, including in China, and relies on biomedical science, genetics, and advanced medical technologies to diagnose, treat, and prevent illness [11]. In contrast, TCM, rooted in ancient China, follows a distinct philosophy with unique principles, like Yin-Yang and the Five Elements, and employs practices such as acupuncture and herbal remedies [12]. The theoretical and diagnostic foundation of TCM is difficult to interpret through the framework of Western medicine anatomy and physiology; it is based on the philosophy, logic, and beliefs of a distinct civilization, offering a view of health and disease that differs from modern scientific thinking [13]. Ancient texts such as *Huangdi Nei Jing* and *Shanghan Lun* remain core guides for TCM students today [14]. TCM was categorized as a kind of complementary and alternative medicine by the World Health Organization and has been adopted by over 100 countries [15]. In 2020, the United States had over 30,000 licensed acupuncturists, while Germany and Australia also had thriving TCM practitioner communities, with more than 3000 and 4000 practitioners, respectively [12]. In 2020, there were 682,770 registered TCM clinicians, accounting for 16.7% of all clinicians, in mainland China, who provided medical services for over 1 billion outpatients and 35 million hospitalizations [16]. Considering the massive medical work undertaken by TCM clinicians, it is important to understand their influenza vaccination preferences.

We conducted a nationwide survey using a discrete choice experiment (DCE) among both TCM and Western medicine clinicians from January to May 2022 in China, to capture their stated preferences regarding influenza vaccination programs. This study’s objectives were: (1) to investigate how different characteristics of influenza vaccination programs influenced clinicians’ vaccination decisions, including TCM and Western medicine clinicians; (2) to estimate the expected influenza vaccine coverage and identify the optimal influenza vaccination program among TCM and Western medicine clinicians; and (3) to identify different subgroups that share certain outward preferences using latent class analysis (LCA).

## Methods

### Participants

**Figure 1.**
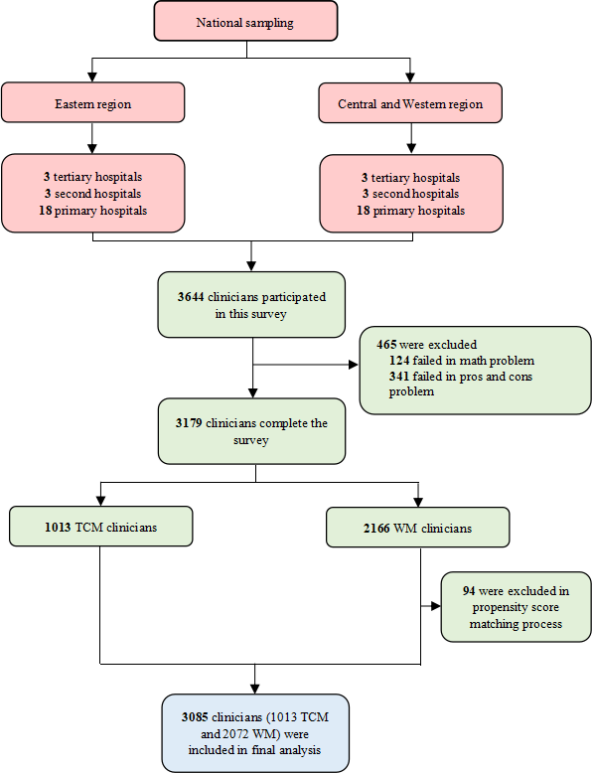
Sample flow chart. TCM: traditional Chinese medicine; WM: Western medicine.

Clinicians (both TCM and Western medicine) holding a current practicing license in a hospital or community health service center, as of the date of survey administration, were included as potential participants. Data were collected through stratified cluster sampling between January 10 and May 10, 2022. In China, the coastal region (Eastern) and inland region (Central and Western) were stratified according to geographical location and socioeconomic development. Three tertiary hospitals, 3 secondary hospitals, and 18 primary hospitals were selected as survey spots in both the Eastern region and the Central and Western regions. The names and locations of these tertiary and secondary hospitals are listed in Table S1 in Multimedia Appendix 1. The primary hospitals were selected with the help of local health bureaus. They selected these hospitals through internal networks, and the name of each primary hospital was unknown to the researchers. We confirmed the location of primary hospital clinicians using their internet protocol address when they accessed the online questionnaire. The geographic distribution of all participants is presented in Figure S1 in Multimedia Appendix 1. Each hospital established a messaging group through WeChat (a free messaging and calling app) that included all the clinicians who worked in the hospital. Hospital administrative staff and local health bureaus delivered the online questionnaire link to these WeChat groups. We provided a brief statement for every participant before they answered the questionnaire to introduce the objective of our survey and to obtain informed consent. All participants were anonymized. Each WeChat account was allowed to submit only 1 online questionnaire. In China, real-name registration is required to apply for a phone number, and each phone number can be linked to only 1 WeChat account. Thus, we can reasonably assume that each individual could complete only 1 questionnaire. Participants who completed the questionnaire and correctly answered 2 quality control questions (a simple math problem and a pros-and-cons question where 1 choice is better than the other) were rewarded with a modest monetary incentive. Before the questionnaire was programmed, it was pilot tested by experts as well as 30 clinicians, who were contacted through our personal network, and adapted based on the feedback received. After sampling, we adjusted the Western medicine clinician sample using the propensity score matching method to ensure comparability between TCM clinicians and Western medicine clinicians (Figure 1).

### Demographic Variables

We used a 2-part questionnaire with demographic questions and DCE options. The demographic variables assessed are listed in Textbox 1.

Textbox 1. **Demographic variables assessed.**

- Age in years, divided into groups (≤29, 30–39, 40–49, or ≥50 years)
- Sex (male or female)
- Educational level (junior college degree or lower, bachelor’s degree, postgraduate degree, or doctorate)
- Medical license type (traditional Chinese medicine, Western medicine, or traditional Chinese medicine integrated with Western medicine)
- Annual personal income (CNY <¥100,000, ¥100,000-¥200,000, or >¥200,000; US <$13,649.18, $13,649.18-$27,298.35, or >$27,298.35, respectively)
- Length of time practicing medicine, in years (≤3 , 4-9, 10-19, 20-29, or ≥30 years)
- Professional (job) title (resident, fellow, or attending)
- Hospital level (primary, secondary, or tertiary)

### Discrete Choice Experiment

A DCE is a technique used widely in the health care sector that elicits people’s preferences by prompting them to make specific choices [17]. In this study, participants were asked to select a series of preferable vaccination programs. Each program was defined by a set of attributes, such as cost of the vaccine and risk of mild adverse events, and the variation in each attribute was referred to as a level. By making a series of choices between a vaccination program with one set of attribute levels and another with alternative attribute levels, participants implicitly revealed the degree to which each attribute is more important to them and the value they place on each attribute level.

This DCE was designed according to health-based principles, ranging from technicalities, such as design efficiency, to participant-centered considerations, such as checking if experimental tasks were as clear as possible to participants. In our survey, individuals made 8 choices, and for each choice they needed to choose a preferred influenza vaccination program between 2 alternative influenza vaccination programs. The influenza vaccination programs were described by attributes and levels representing different characteristics of vaccination programs.

### Attributes and Levels

Five attributes described the characteristics of vaccination programs in the choice tasks, summarized in Table 1. We selected attributes and levels including: variable cost of vaccine (CNY ¥0; CNY ¥50 [approximately US $7]; or CNY ¥100 [approximately US $14]), variable vaccine effectiveness against infection (20%, 50%, or 80%), variable risk of mild adverse events such as redness, swelling, or pain at the injection site (1%, 3%, or 5%), different vaccination campaign strategies (individual appointment or vaccination in a workplace setting), and different workplace attitudes (no notice or encouragement of vaccination).

**Table 1.** Attributes and levels of discrete choice experiment.

Attribute	Levels
1. Cost of vaccine	CNY ¥0 CNY ¥50 (approximately $7 USD) CNY ¥100 (approximately $15 USD)
2. Vaccine effectiveness against infection	Prevent 20% infection Prevent 50% infection Prevent 80% infection
3. Risk of mild adverse events (such as redness, swelling, or pain at the injection site)	1% risk 3% risk 5% risk
4. Vaccination campaign strategies	Individual appointment Vaccination in a workplace setting
5. Workplace attitudes	No notice Encouragement of vaccination

We selected attributes and levels related to influenza vaccination based on several sources of evidence: a rapid review of existing research [18-21], one-on-one interviews with 4 TCM clinicians and 4 Western medicine clinicians, and a consultation with subject matter experts. An orthogonal experimental design was used to reduce these choice tasks down to 8 choice tasks with 16 hypothetical vaccination programs. Levels were randomly assigned with equal probabilities and repeatedly recombined, with all combinations being plausible and realistic. Priors were obtained from a pilot study of 30 clinicians. Each clinician answered 8 choice tasks, reporting no understanding problem and respondent fatigue. The full choice tasks, as presented to respondents, are shown in Table S2 in Multimedia Appendix 1.

### Statistical Analyses

The rule of thumb, as proposed by Johnson and Orme [22], suggests that the sample size required for the main effects of a DCE analysis depends on the number of choice tasks (*t*), the number of alternatives for each choice task (*a*), and the largest number of levels for any of the attributes (*c*), according to the following equation [23]:


N§gt;1000c/(t×a)

For our study, there were 8 choice tasks; each choice task had 2 alternatives, and the largest number of levels was 3 for any of the attributes. Our sample, which exceeded the minimum sample size calculated, of approximately at least 376 clinicians (or 188 of both TCM and Western medicine clinicians), was sufficient for the purposes of our study.

Sex, age groups, and hospital levels were identified as confounders in propensity score matching, and the propensity score of the Western medicine sample and TCM sample was calculated. We then used a nearest-neighbor matching algorithm to match 3 Western medicine clinicians with 1 TCM clinician based on the propensity score. Descriptive statistics were used to summarize characteristics of TCM and Western medicine clinicians. For categorical variables, frequencies were reported, and Pearson *χ*² tests were used to test for differences between the 2 groups. For continuous variables, mean and standard deviation were reported after Shapiro-Wilk tests indicated that the continuous variables were normally distributed. We used *t* tests to compare differences across the 2 groups.

To estimate the relative impact of influenza vaccine attributes in the DCE, we conducted a mixed logit regression model (MLM) to compute preference weight. An MLM is based on the assumption that random error has a normal distribution, taking heterogeneity as well as correlation between the choice task completed by each participant into account. We validated the linear continuous effects of chosen attributes, and the variables of cost, vaccine efficacy, and risk of mild adverse events were considered as continuous, while the vaccination campaign strategies and workplace attitudes were considered as categorized in the analysis. The MLM allowed for the calculation of compensatory effects across any 2 attributes. For example, a 5% decrease of risk of mild adverse events can compensate the negative impact of a 10% reduction of vaccine effectiveness. The willingness to pay (WTP) refers to the compensatory effect between cost and any other attribute. We calculated the expected vaccine coverage for the base case and the change of coverage when the level of one attribute was changed.

Subsequently, we conducted an LCA to classify individuals based on their preference characteristics; an individual was assigned to the class with the highest posterior probability. For example, the LCA presented that 31.1% (644/2072) of Western medicine clinicians preferred free vaccinations over any other attribute. Our choice in the number of subgroups was based on model fit and interpretability of results. We compared classes across demographic characteristics among the estimated classes using *χ*^*2*^ tests. The preference subgroups were named based on the most preferred attribute levels in each class.

All analyses were based on 2-sided *P* values, with *P*<.05 indicating statistical significance. Stata (Version 16.0, Stata Corp LLC) was used for analysis.

### Ethical Considerations

The Zhejiang Chinese Medical University Ethics Committee reviewed and approved this protocol (no. 20221021‐1). Prior to participation, all study participants were required to sign an informed consent form, thereby confirming their voluntary engagement in the survey process. The study data were anonymized. All participants who completed the questionnaire and successfully submitted it received a reward of CNY ¥20 (US $2.7), distributed via WeChat.

## Results

### Demographics

A total of 3644 clinicians from 6 tertiary hospitals, 6 secondary hospitals, and 36 primary hospitals were invited to participate in this survey. Among them, 559 (15.3%) were ultimately excluded for failing the basic math tests (n=124, 3.4%) or the pros-and-cons test (n=341, 9.4%) or in the post-hoc during propensity score matching (n=94, 2.6%). Finally, 3085 subjects, consisting of 1013 (32.8%) TCM clinicians and 2072 (67.2%) Western medicine clinicians, were included in the analysis (Figure 1).

The sample matching adjustment achieved the expected distributions of gender, age groups, and hospital level (Figures S2-S4 in Multimedia Appendix 1). Educational attainment showcased a significant discrepancy, with a notable proportion of Western medicine clinicians holding a bachelor’s degree (1106/2072, 53.4%) compared to TCM clinicians (409/1013, 40.4%). Conversely, a higher percentage of TCM clinicians had attained a master’s degree (274/1013, 27%) or a doctorate (110/1013, 10.9%) compared to their Western medicine counterparts. In terms of annual personal income, a higher percentage of TCM clinicians reported incomes of CNY ¥100,000‐¥200,000 (US$13,649.18-$27,298.35) (345/1013, 34.1%) compared to Western medicine clinicians (570/2072, 27.5%). Conversely, a higher proportion of Western medicine clinicians reported incomes above CNY ¥200,000 (US $27,298.35) (413/2072, 19.9%) compared to TCM clinicians (133/1013, 13.2%). In terms of professional title, 50.4% (511/1013) of TCM clinicians held the title of resident compared to Western medicine clinicians (916/2072, 44.2%). Conversely, a higher proportion of Western medicine clinicians held the title of fellow (827/2072, 39.9%) compared to TCM clinicians (346/1013, 34.2%). For the length of time practicing medicine, there was no significant difference between TCM and Western medicine clinicians (*P*=.44). (Table 2).

**Table 2.** Demographic data of clinicians practicing traditional Chinese medicine (TCM) and Western medicine (WM) between January and May 2022 in China.

	TCM clinicians (n=1013), n (%)	WM clinicians (n=2072), n (%)	*P* value
Sex			.31
Male	420 (41.5)	899 (43.4)	
Female	593 (58.5)	1173 (56.6)	
Age group (year)			.63
0-29	319 (31.5)	618 (29.8)	
30-39	431 (42.5)	893 (43.1)	
40-49	183 (18.1)	407 (19.6)	
≥50	80 (7.9)	154 (7.5)	
Hospital level			.55
Primary	295 (29.1)	637 (30.7)	
Secondary	240 (23.7)	498 (24)	
Tertiary	478 (47.2)	937 (45.2)	
Educational level			<.001
Junior college degree or lower	220 (21.7)	584 (28.2)	
Bachelor’s degree	409 (40.4)	1106 (53.4)	
Master’s degree	274 (27)	289 (13.9)	
Doctorate	110 (10.9)	93 (4.5)	
Annual personal income (CNY^a^)			<.001
0-100,000	535 (52.8)	1089 (52.6)	
100,000-200,000	345 (34.1)	570 (27.5)	
>200,000	133 (13.2)	413 (19.9)	
Length of time practicing medicine (year)			.44
0-3	287 (28.3)	543 (26.2)	
4–9	331 (32.7)	677 (32.7)	
10–19	242 (23.9)	540 (26.1)	
20–29	106 (10.5)	232 (11.2)	
≥30	47 (4.6)	80 (3.9)	
Professional title			.003
Resident	511 (50.4)	916 (44.2)	
Fellow	346 (34.2)	827 (39.9)	
Attending	156 (15.4)	329 (15.9)	

a CNY: Chinese yuan. CNY ¥1=US $0.14.

### Preference Weight and Compensatory Effects (Including WTP)

All attributes and levels included were significantly associated with clinicians’ preferences for influenza vaccination programs. Both TCM and Western medicine clinicians’ preferences for influenza vaccination programs increased with higher vaccine effectiveness, with the vaccination campaign strategies changing from “individual appointment” to “vaccination in a workplace setting,” or with the workplace attitude changing from “no notice” to “encouraging of vaccination.” Nevertheless, the increase in the risk of mild adverse events and cost could undermine the utility of vaccination towards these 2 groups. The WTPs of TCM clinicians were calculated and showed that each 1% increase in risk of mild adverse events could be compensated by a CNY ¥6.97 (US $1.37) decrease of cost. Similarly, changing the mode of administration from “individual appointment” to “vaccination in a workplace setting” could compensate for the negative effect of a CNY ¥78.84 (US $15.44) increase of cost. Compensatory effects can be calculated between any attributes; for example, a hypothetical 5% increase in risk of mild adverse events could be compensated by a simultaneous hypothetical 13.7% increase in vaccine effectiveness. WTP and the compensatory effect of Western medicine clinicians were calculated in the same way. The preference weights and WTP of attributes and levels of the 2 groups are shown in Table S3 in Multimedia Appendix 1.

### Comparison of Preference Weights Among TCM and Western Medicine Clinicians

**Figure 2.**
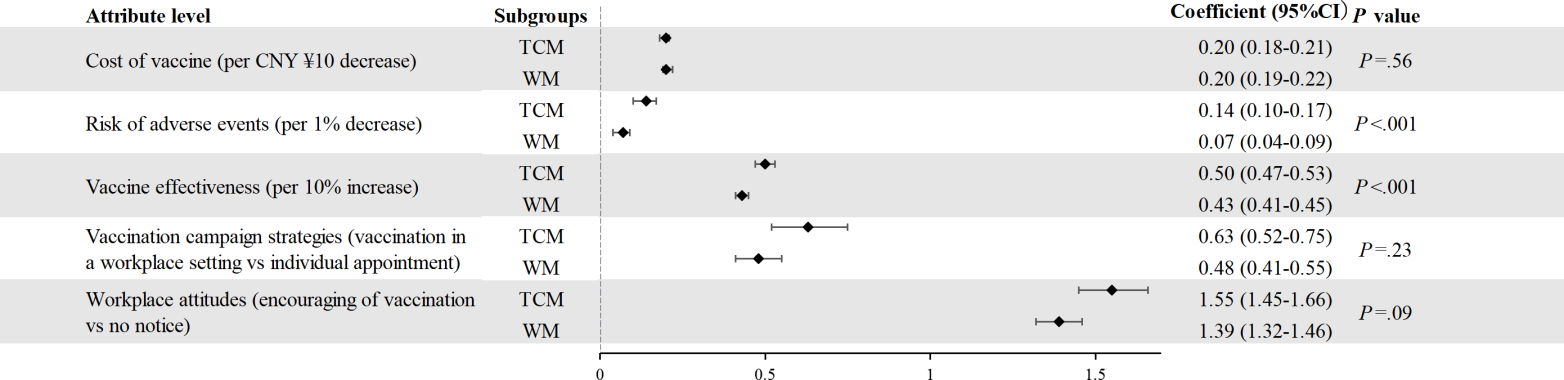
Comparison of preferences in clinicians practicing TCM and WM between January and May 2022 in China. TCM: traditional Chinese medicine; WM: Western medicine.

Significant differences were noted for the coefficients of the risk of mild adverse events and vaccine effectiveness between clinicians practicing TCM and Western medicine. TCM clinicians (odds ratio [OR] 0.14, 95% CI 0.10‐0.17) expressed a stronger preference for an influenza vaccination program per 1% decrease of mild adverse events risk than Western medicine clinicians (OR 0.07, 95% CI 0.04‐0.09; *P*<.001). Similarly, TCM clinicians (OR 0.50, 95% CI 0.47‐0.53) expressed a stronger preference for an influenza vaccination program per 10% increase of vaccine effectiveness than Western medicine clinicians (OR 0.43, 95% CI 0.41‐0.45; *P*<.001). Other attributes (cost, vaccination campaign strategies, and workplace attitudes) did not present significant differences between the 2 groups (Figure 2).

### Expected Influenza Vaccine Coverage Under Various Influenza Vaccination Programs

**Figure 3.**
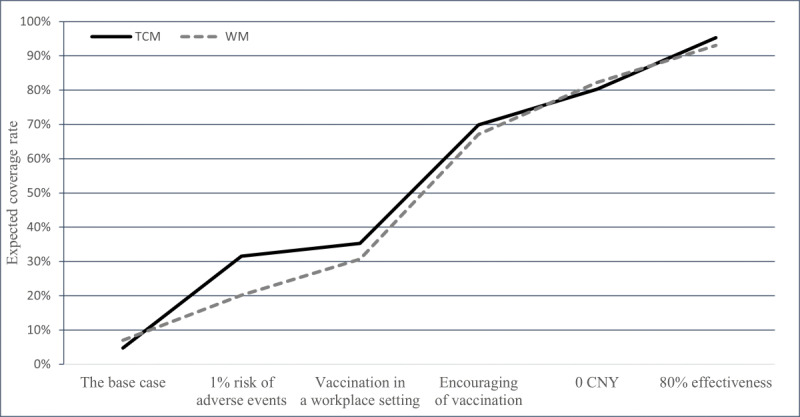
Changes in expected coverage rate, as attributes in influenza vaccination programs improve, between January and May 2022 in China. We set the base case as having a cost of CNY ¥100 (US $13.64), 5% risk of mild adverse events, 20% effectiveness, vaccination by individual arrangement, and no notice from the workplace. TCM: traditional Chinese medicine; WM: Western medicine; CNY: Chinese yuan.

We set the base case as having a cost of CNY ¥100 (US $13.64), 5% risk of mild adverse events, 20% effectiveness, vaccination by individual arrangement, and no notice from the workplace. The expected coverage of the base case to TCM clinicians was 4.75% and to Western medicine clinicians was 6.99%. Expected coverage increased by approximately 30% with a reduction in risk of mild adverse events from 5% to 1%; greater coverage increases could be achieved by either enhancing effectiveness from 20% to 80% or reducing costs from CNY ¥100 to CNY ¥0. An approximate hypothetical 60% increase could be obtained when the attitude of the workplace changed from “no notice” to “encouraging of vaccination,” and there was an approximate hypothetical 35% increase when vaccination campaign strategies changed from “individual appointment” to “vaccination in a workplace setting.” The changing of expected coverage due to improvements in the influenza vaccination program is depicted in Figure 3 (additional details available in Table S4 in Multimedia Appendix 1).

### Preference of Different Subgroups

Table 3 details the demographic composition variations across various subgroups, while the fitting and categorization process of these subgroups is recorded in Table S5 in Multimedia Appendix 1. In the entire sample, about 30% (946/3085) of clinicians preferred free vaccinations, while approximately 26% (819/3085) comprehensively considered all attributes except for vaccination campaign strategies. Notable trends were observed within 2 specific subgroups: clinicians preferring free vaccinations and those preferring vaccinations in a workplace setting. Among clinicians with different medical licenses, TCM practitioners exhibited a lower preference for free vaccinations (302/1013, 29.8%) compared to Western medicine clinicians (644/2072, 31.1%) and a higher preference for workplace vaccinations (186/1013, 18.4%) compared to Western medicine clinicians (351/2072, 17%) (*P*=.03). Differences were also noted across sex, with female clinicians indicating a lower preference for free vaccinations (533/1766, 30.2%) compared to male clinicians (414/1319, 31.3%) and a higher preference for workplace vaccinations (317/1766, 18%) compared to male clinicians (219/1319, 16.6%) (*P*=.04). Educational level revealed a U-shaped relationship, where the highest preference for free vaccinations was among those with junior college degrees or lower (270/804, 33.6%); however, this preference decreased significantly among those with postgraduate degrees (158/563, 28%; *P*<.001). Conversely, there was an inverted U-shaped relationship between educational level and preference for vaccinations in a workplace setting. A strong monotonic relationship was present between hospital level and clinicians’ preference for free vaccinations, with 32.5% (303/932) of primary hospital workers preferring free vaccinations compared to 29.4% (416/1415) of those in tertiary hospitals (*P*<.001). On the other hand, clinicians in tertiary hospitals (262/1415, 18.5%) significantly preferred vaccinations in a workplace setting compared to those in primary (145/932, 15.6%) or secondary hospitals (131/738, 17.7%) (*P*<.001). No significant differences were found based on age group, annual personal income, length of time practicing medicine, or professional title.

**Table 3.** Demographic characteristics by influenza vaccination preference from a latent class analysis between January and May 2022 in China.

Demographics and categories	Prefer encouragement of vaccinations in a workplace setting, n (%)	Prefer free vaccinations, n (%)	Prefer higher vaccine effectiveness, n (%)	Consider all attributes except vaccination campaign strategies, n (%)	Prefer vaccinations in a workplace setting, n (%)	*P* value
Medical practicing license						.03
TCM^a^ (n=1013)	144 (14.2)	302 (29.8)	113.46 (11.2)	267 (26.4)	186 (18.4)	
WM^b^ (n=2072)	300 (14.5)	644 (31.1)	223.78 (10.8)	552 (26.7)	351 (17)	
Age group (year)						.54
0-29 (n=937)	137 (14.6)	292 (31.2)	102 (11)	242 (25.8)	164 (17.5)	
30-39 (n=1324)	188 (14.2)	408 (30.8)	144.32 (10.9)	357 (27)	226 (17.1)	
40-49 (n=590)	87 (14.7)	173 (29.3)	64.31 (10.9)	158 (26.7)	109 (18.4)	
≥50 (n=234)	34 (14.5)	73 (31.2)	24.57 (10.5)	63 (27)	39 (16.8)	
Sex						.04
Male (n=1319)	193 (14.6)	414 (31.3)	142.45 (10.8)	351 (26.6)	219 (16.6)	
Female (n=1766)	253 (14.3)	533 (30.2)	193 (11)	470 (26.6)	317 (18)	
Educational level						<.001
Junior college degree or lower (n=804)	118 (14.7)	270 (33.6)	85.22 (10.6)	211 (26.3)	119 (14.8)	
Bachelor’s degree (n=1515)	218 (14.4)	459 (30.3)	166 (11)	402 (26.6)	270 (17.8)	
Postgraduate degree (n=563)	80 (14.2)	158 (28)	63.62 (11.3)	151 (26.9)	110 (19.6)	
Doctorate (n=203)	28 (14)	59 (29.3)	21.92 (10.8)	54 (26.7)	39 (19.2)	
Annual personal income (CNY^c^)						.63
0-100,000 (n=1624)	235 (14.5)	505 (31.1)	175.39 (10.8)	431 (26.5)	277 (17)	
100,000-200,000 (n=915)	131 (14.3)	278 (30.4)	99.74 (10.9)	243 (26.6)	163 (17.8)	
>200,000 (n=546)	78 (14.2)	163 (29.9)	60.61 (11.1)	145 (26.6)	99 (18.2)	
Length of time practicing medicine, in years						.79
0-3 (n=830)	120 (14.5)	262 (31.6)	91.3 (11)	221 (26.6)	135 (16.4)	
4–9 (n=1008)	147 (14.6)	305 (30.3)	107.86 (10.7)	265 (26.3)	182 (18.1)	
10–19 (n=782)	111 (14.2)	236 (30.2)	86 (11.1)	210 (26.8)	139. (17.8)	
20–29 (n=338)	49 (14.4)	103 (30.5)	37.52 (11.1)	91 (27)	58 (17.1)	
≥30 (n=127)	18 (14.3)	39 (30.7)	13.21 (10.4)	34 (26.9)	22 (17.7)	
Professional (job) title						.28
Residents (n=1427)	207 (14.5)	448 (31.4)	154.12 (10.8)	378 (26.5)	240 (16.8)	
Fellows (n=1173)	167 (14.2)	354 (30.2)	129.03 (11)	314 (26.8)	209 (17.8)	
Attending (n=485)	71 (14.6)	144 (29.7)	53.84 (11.1)	128 (26.3)	89 (18.3)	
Hospital level						<.001
Primary (n=932)	133 (14.3)	303 (32.5)	99.72 (10.7)	251 (26.9)	145 (15.6)	
Secondary (n=738)	107 (14.5)	227 (30.7)	80.44 (10.9)	193 (26.2)	131 (17.7)	
Tertiary (n=1415)	204 (14.4)	416 (29.4)	157.07 (11.1)	376 (26.6)	262 (18.5)	

a TCM: traditional Chinese medicine.

b WM: Western medicine.

c CNY: Chinese yuan. CNY ¥1=US $0.14.

## Discussion

### Principal Findings

We found that (1) TCM clinicians were more sensitive to changes to the risk of mild adverse events and vaccine effectiveness than Western medicine clinicians; (2) decreasing the price of influenza vaccinations, offering convenient vaccinations in workplace settings, and issuing notifications encouraging clinicians to get vaccinated could increase influenza vaccination coverage of both groups; and (3) compared to Western medicine clinicians, a larger proportion of TCM clinicians preferred vaccinations in workplace settings over any other attribute.

The findings indicate a higher probability of vaccines being received when paired with increased vaccine effectiveness and decreased likelihood of vaccine-related adverse events, which aligns with prior research conducted through questionnaire surveys [24]. In our survey, TCM clinicians placed significantly higher preference weights on the risk of mild adverse events and vaccine effectiveness, indicating that fluctuations in the safety and effectiveness of influenza vaccines could have a greater impact on them. A qualitative study shows that patients find vaccination information transmitted by complementary and alternative medicine providers to be more understandable, useful, and trustworthy [25]. A TCM clinician who receives and shares information regarding defective vaccine incidents could cause a chain reaction in the broad population. A systematic review indicated that the provision of free vaccinations, easy access to vaccinations, and modification through educational activities and reminders can effectively increase the influenza vaccine uptake [26]. In the simulation scenario, decreasing the price of the influenza vaccination from CNY ¥100 (US $13.64) to being free resulted in a significant increase in projected coverage. Offering free vaccinations to clinicians can provide additional benefits, such as mitigating absenteeism during the influenza season and preventing hospital-acquired influenza infections. We also found that other workplace-based interventions, such as providing vaccination opportunities at the workplace and promoting vaccination initiatives more broadly, demonstrated a favorable effect on the projected uptake of influenza vaccine. Given the feasibility and efficacy of these measures, we propose that health care facilities should (1) provide free annual influenza vaccinations, (2) furnish accessible vaccination services for employees, and (3) issue notifications encouraging clinicians to avail themselves of vaccination opportunities. In our LCA, the majority of clinicians prioritized free vaccinations, followed by a substantial group who took into account all attributes except vaccination campaign strategies when deciding whether to accept an influenza vaccination program. The results of our survey suggest that a higher proportion of TCM clinicians favored workplace-based vaccinations over any other attribute, whereas a greater number of Western medicine clinicians preferred free vaccinations over any other attribute. This finding can lend support to the creation of a vaccination policy that is more flexible and adaptable. Sex, educational level, and hospital level were all associated with latent classification. Among these considerations, the most significant distinction emerged between clinicians who preferred free vaccinations most and those who preferred vaccinations in a workplace setting most. This differentiation suggests that the other 3 categories (favoring workplace encouragement of vaccinations, prioritizing higher vaccine effectiveness, and considering attributes except vaccination campaign strategies) were evenly distributed across all demographics in our study. As a result, measures like releasing notices in health care workplaces to encourage clinicians to get vaccinated may achieve similar effects across all groups of Chinese clinicians. A previous study showed that women’s perceptions of inflation (consumer prices increasing) were higher than men’s, but fewer female clinicians preferred free vaccinations over vaccinations in a workplace setting, compared to male clinicians in our study [27]. A policy offering free vaccinations may hold greater appeal for clinicians with lower levels of education (bachelor’s degree or below) or those employed in primary health care institutions. Conversely, providing vaccinations in a workplace setting was found to be more favorable among clinicians with higher levels of education (postgraduate degree or doctorate) or those working in tertiary hospitals. In China, clinicians employed in tertiary hospitals serve as the cornerstone of the nation’s health care provision, handling extensive medical responsibilities that may make it challenging for them to get vaccinated during their private leisure time. Consequently, providing convenient vaccination services for clinicians should be prioritized as a primary measure for these tertiary hospitals.

### Limitations

Our study is subject to several limitations. First, our sample of clinicians did not perfectly mirror the sociodemographic characteristics of the entire nation. Additionally, there may be biases between individuals who completed the survey and those who did not. Second, as a cross-sectional study, potential recall bias is inevitable. Moreover, DCEs are susceptible to hypothetical bias, meaning that responses in surveys may not perfectly align with real-life behaviors [28]. This discrepancy could limit the accuracy of measured preferences. To mitigate this, we designed the experiment based on real-world influenza vaccination programs and ensured that the details and definitions of each attribute were comprehensible to participants.

### Conclusions

In summary, while changes in mild adverse event rates and vaccine effectiveness had a greater impact on TCM clinicians, other attributes, such as workplace-provided free vaccinations, issuing notifications to encourage vaccinations, and providing accessible vaccination services, are practical methods to increase the uptake of influenza vaccination programs among both groups. Policymakers can utilize these insights to formulate flexible intervention measures that facilitate greater accessibility of influenza vaccination programs for clinicians, especially TCM clinicians, worldwide.

## Supplementary material

10.2196/63314 Multimedia Appendix 1 Supplementary tables and figures.

## Abbreviations

DCE: discrete choice experiment
HCW: health care worker
MLM: mixed logit regression model
OR: odds ratio
TCM: traditional Chinese medicine
WTP: willingness to pay
