# Supplementary material for: Identifying Preferred Features of Influenza Vaccination Programs Among Chinese Clinicians Practicing Traditional Chinese Medicine and Western Medicine: Discrete Choice Experiment
Source: JMIR Public Health Surveill. 2025 Jan 20;11:e63314. doi: 10.2196/63314 (PMC11769775; doi:10.2196/63314)
Supplement: Multimedia Appendix 1 [file publichealth-v11-e63314-s001.docx]

CONTENTS

[Figure S1: Density of all the participants included in national sampling between January – May 2022 in China 2](#_Toc187323771)

[Figure S2. Comparison of the sex distributions of traditional Chinese medicine and western medicine clinicians between January – May 2022 in China 3](#_Toc187323772)

[Figure S3. Comparison of the age groups distributions of traditional Chinese medicine and western medicine clinicians between January – May 2022 in China 4](#_Toc187323773)

[Figure S4. Comparison of the hospital level distributions of traditional Chinese medicine and western medicine clinicians between January – May 2022 in China 5](#_Toc187323774)

[Table S1. The list of hospitals selected as survey spot in the study. 6](#_Toc187323775)

[Table S2. The 8 choice tasks with 16 hypothetical vaccination programs of discrete choice experiment 7](#_Toc187323776)

[Table S3. Influenza vaccination preference weight and willing to pay of clinicians practicing in traditional Chinese medicine and western medicine between January – May 2022 in China 9](#_Toc187323777)

[Table S4. Change of expected coverage rate with the changes in attribute levels of clinicians practicing in traditional Chinese medicine and western medicine between January – May 2022 in China 10](#_Toc187323778)

[Table S5. Model fit for different numbers of classes 11](#_Toc187323779)

# Figure S1: Density of all the participants included in national sampling between January – May 2022 in China


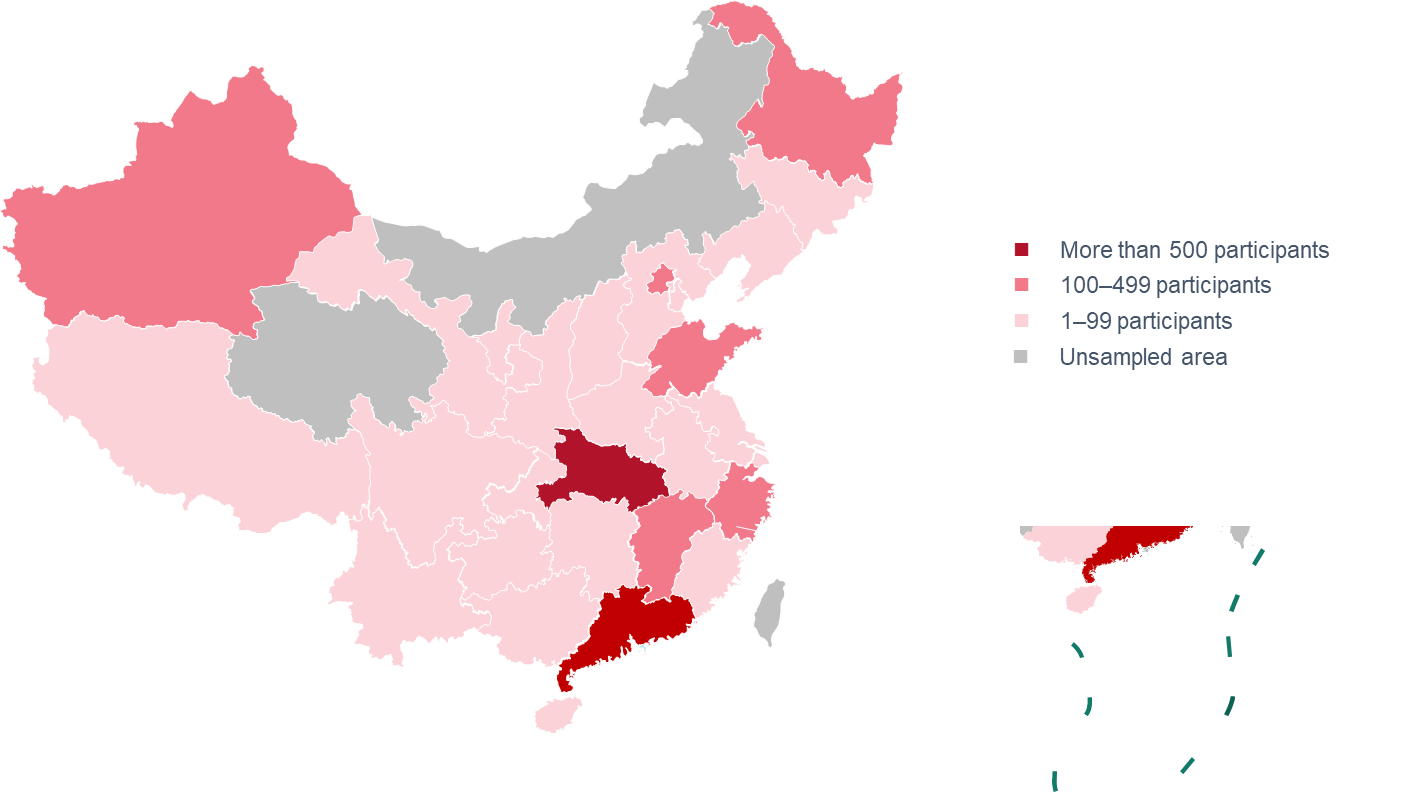


# Figure S2. Comparison of the sex distributions of traditional Chinese medicine and western medicine clinicians between January – May 2022 in China

TCM: traditional Chinese medicine

WM: western medicine

# Figure S3. Comparison of the age groups distributions of traditional Chinese medicine and western medicine clinicians between January – May 2022 in China

TCM: traditional Chinese medicine

WM: western medicine

# Figure S4. Comparison of the hospital level distributions of traditional Chinese medicine and western medicine clinicians between January – May 2022 in China

P=0.55

TCM: traditional Chinese medicine

WM: western medicine

# Table S1. The list of hospitals selected as survey spot in the study.

| Region | Hospital level | Name of hospital (Province) |
| --- | --- | --- |
| Eastern region | Tertiary hospital | Huazhong University of Science and Technology Union Shenzhen Hospital (Guangdong) |
|  |  | Zhejiang Hospital of Traditional Chinese Medicine (Zhejiang) |
|  |  | Dongzhimen Hospital (Beijing) |
|  | Secondary hospital | Anqiu hospital of Traditional Chinese Medicine (Shandong) |
|  |  | Shenzhen Longgang district Orthopedic Hospital (Guangdong) |
|  |  | Hainan Hospital of Integrated Chinese and Western Medicine (Hainan) |
|  | Primary hospital | Name unknown |
| Central and western region | Tertiary hospital | Macheng people’s hospital (Hubei) |
|  |  | Xinjiang first people’s hospital (Xinjiang) |
|  |  | Harbin First Specialty Hospital (Heilongjiang) |
|  | Secondary hospital | Macheng maternal and Child Health Hospital (Hubei) |
|  |  | Dexing hospital of traditional Chinese medicine (Jiangxi) |
|  |  | Hongan No.2 people’s hospital (Hubei) |
|  | Primary hospital | Name unknown |

Note: The primary hospitals were selected with the help of local health bureaus. They selected these hospital through internal network and the name of each primary hospital was unknown to researchers.

# Table S2. The 8 choice tasks with 16 hypothetical vaccination programs of discrete choice experiment

| Choice task 1 | | |
| --- | --- | --- |
|  | Vaccination program A | Vaccination program B |
| Risk of mild adverse events | 5% | 5% |
| Vaccine effectiveness | 80% | 50% |
| Vaccination campaign strategies | Vaccination in a workplace setting | Individual appointment |
| Workplace attitudes | Encourage vaccination | No-notice |
| Cost of vaccine | 0 CNY | 0 CNY |
| Which program do you prefer? □ □ | | |
|  | | |
| Choice task 2 | | |
|  | Vaccination program A | Vaccination program B |
| Risk of mild adverse events | 3% | 1% |
| Vaccine effectiveness | 20% | 50% |
| Vaccination campaign strategies | Individual appointment | Individual appointment |
| Workplace attitudes | Encourage vaccination | Encourage vaccination |
| Cost of vaccine | 0 CNY | 50 CNY |
| Which program do you prefer? □ □ | | |
|  | | |
| Choice task 3 | | |
|  | Vaccination program A | Vaccination program B |
| Risk of mild adverse events | 1% | 1% |
| Vaccine effectiveness | 20% | 20% |
| Vaccination campaign strategies | Individual appointment | Individual appointment |
| Workplace attitudes | Encourage vaccination | No-notice |
| Cost of vaccine | 0 CNY | 50 CNY |
| Which program do you prefer? □ □ | | |
|  | | |
| Choice task 4 | | |
|  | Vaccination program A | Vaccination program B |
| Risk of mild adverse events | 1% | 1% |
| Vaccine effectiveness | 80% | 50% |
| Vaccination campaign strategies | Vaccination in a workplace setting | Vaccination in a workplace setting |
| Workplace attitudes | No-notice | Encourage vaccination |
| Cost of vaccine | 0 CNY | 100 CNY |
| Which program do you prefer? □ □ | | |
|  | | |
| Choice task 5 | | |
|  | Vaccination program A | Vaccination program B |
| Risk of mild adverse events | 5% | 1% |
| Vaccine effectiveness | 20% | 80% |
| Vaccination campaign strategies | Vaccination in a workplace setting | Individual appointment |
| Workplace attitudes | Encourage vaccination | No-notice |
| Cost of vaccine | 0 CNY | 0 CNY |
| Which program do you prefer? □ □ | | |
|  | | |
| Choice task 6 | | |
|  | Vaccination program A | Vaccination program B |
| Risk of mild adverse events | 3% | 3% |
| Vaccine effectiveness | 50% | 80% |
| Vaccination campaign strategies | Vaccination in a workplace setting | Individual appointment |
| Workplace attitudes | No-notice | Encourage vaccination |
| Cost of vaccine | 0 CNY | 100 CNY |
| Which program do you prefer? □ □ | | |
|  | | |
| Choice task 7 | | |
|  | Vaccination program A | Vaccination program B |
| Risk of mild adverse events | 5% | 1% |
| Vaccine effectiveness | 20% | 20% |
| Vaccination campaign strategies | Individual appointment | Vaccination in a workplace setting |
| Workplace attitudes | No-notice | No-notice |
| Cost of vaccine | 100 CNY | 100 CNY |
| Which program do you prefer? □ □ | | |
|  | | |
| Choice task 8 | | |
|  | Vaccination program A | Vaccination program B |
| Risk of mild adverse events | 1% | 3% |
| Vaccine effectiveness | 20% | 20% |
| Vaccination campaign strategies | Vaccination in a workplace setting | Vaccination in a workplace setting |
| Workplace attitudes | Encourage vaccination | No-notice |
| Cost of vaccine | 0 CNY | 50 CNY |
| Which program do you prefer? □ □ | | |

CNY: Chinese yuan

# Table S3. Influenza vaccination preference weight and willing to pay of clinicians practicing in traditional Chinese medicine and western medicine between January – May 2022 in China

|  | **TCM clinicians** | |  | **WM clinicians** | |
| --- | --- | --- | --- | --- | --- |
|  | **Coefficient (95%CI）** | **Willing to pay** |  | **Coefficient (95%CI)** | **Willing to pay** |
| **Cost of vaccine (per 10 ¥ decrease)** | 0.20 (0.18—0.21) | - |  | 0.20 (0.19—0.22) | - |
| **Risk of adverse events (per 1% decrease)** | 0.14 (0.10—0.17) | 6.97 |  | 0.07 (0.04—0.09) | 3.39 |
| **Vaccine effectiveness (****per 10% increase)** | 0.50 (0.47—0.53) | 25.38 |  | 0.43 (0.41—0.45) | 22.05 |
| **Vaccination campaign strategies** |  |  |  |  |  |
| Individual appointment | Ref. | - |  | Ref. |  |
| Vaccination in a workplace setting | 0.63 (0.52—0.75) | 31.96 |  | 0.48 (0.41—0.55) | 24.63 |
| **Workplace attitude** |  |  |  |  |  |
| No-notice | Ref. | - |  | Ref. | - |
| Encouraging of vaccination | 1.55 (1.45—1.66) | 78.84 |  | 1.39 (1.32—1.46) | 71.06 |
| **Log-likelihood** | -3482.71 |  |  | -8354.75 |  |
| **Number of respondents** | 1013 |  |  | 2072 |  |
| **Number of observations** | 16208 |  |  | 33152 |  |

TCM: traditional Chinese medicine

WM: western medicine

CI: confidence interval

# Table S4. Change of expected coverage rate with the changes in attribute levels of clinicians practicing in traditional Chinese medicine and western medicine between January – May 2022 in China

|  | **TCM clinicians (%, 95%CI)** | **WM clinicians (%, 95%CI)** |
| --- | --- | --- |
| **The base case** | 4.75(4.29-5.21) | 6.99 (6.18-7.79) |
| **Cost of vaccine** reducing from 100 to 0 CNY | 80.27 (78.31-82.23) | 82.20 (81.04-83.78) |
| **Risk of adverse events** reducing from 5% to 1% | 31.54 (27.58-35.50) | 20.16 (18.03-23.33) |
| **Effectiveness** increasing from 20% to 80% | 95.25 (93.86-96.64) | 93.02 (91.46-93.90) |
| **Vaccination campaign strategy** changing from individual appointment to vaccination in workplace setting | 35.25 (32.12-38.38) | 30.62 (28.21-32.31) |
| **Workplace attitude** changing from no-notice to encourage of vaccination | 69.85 (67.86-71.84) | 67.10 (65.71-68.61) |

The base case: We set the base case was with cost 100 CNY, risk of mild adverse events 5%, effectiveness 20%, vaccination by individual arrangement and no-notice from workplace.

TCM: traditional Chinese medicine

WM: western medicine

CNY: Chinese yuan

CI: confidence interval

# Table S5. Model fit for different numbers of classes

| Model | AIC | BIC | aBIC | entropy | LMR  *p* value | ALMR *p* value | BLRT *p* value |
| --- | --- | --- | --- | --- | --- | --- | --- |
| 1-class | 211289.439 | 211354.349 | 211328.926 | - | - | - | - |
| 2-class | 195651.712 | 195789.646 | 195735.621 | 0.982 | 0.0000 | 0.0000 | 0.0000 |
| 3-class | 182328.437 | 182539.395 | 182456.767 | 1.000 | 1.0000 | 1.0000 | 0.0000 |
| 4-class | 168319.942 | 168603.923 | 168492.694 | 1.000 | 0.0000 | 0.0000 | 0.0000 |
| 5-class | 155004.108 | 155361.113 | 155221.282 | 1.000 | 0.0000 | 0.0000 | 0.0000 |
| 6-class | 146659.564 | 147089.592 | 146921.160 | 0.992 | 1.0000 | 1.0000 | 0.0000 |

AIC: Akaike information criterion

BIC: Bayesian information criterion

aBIC: adjusted Bayesian information criterion

LMR LR: Lo-Mendell-Rubin likelihood ratio test

ALMR LR: adjusted Lo-Mendell-Rubin likelihood ratio test

BLRT: bootstrap likelihood ratio test
